# An unusual presentation of leishmaniasis in a human immunodeficiency virus-positive individual

**DOI:** 10.1099/jmmcr.0.005011

**Published:** 2016-02-05

**Authors:** Marijo S. Roiko, Bryan H. Schmitt, Ryan F. Relich, Thomas L. Meyer, Shanxiang Zhang, Thomas E. Davis

**Affiliations:** ^1^​ Department of Pathology and Laboratory Medicine, Indiana University School of Medicine, Indianapolis, IN 46202, USA; ^2^​ Division of Infectious Disease, Indiana University Health, Indianapolis, IN 46202, USA

**Keywords:** condyloma, HAART, HIV, *Leishmania donovani*, leishmaniasis

## Abstract

**Introduction:**

Leishmaniasis is a neglected tropical disease caused by vector-borne protozoa of the genus *Leishmania*. Cutaneous and mucocutaneous forms result in disfiguration or mutilation, whilst visceral leishmaniasis (VL) affects multiple organs and is fatal if untreated. Notably, *Leishmania* are capable of establishing a chronic infection, which may reactivate years after initial infection when the host becomes immune-suppressed.

**Case Presentation:**

A 24-year-old human immunodeficiency virus (HIV)-positive male presented for excision of anal condylomas. At the time of his current condyloma excision, the patient had no additional symptoms or cutaneous findings, but was noted to have been only intermittently compliant with his antiretroviral therapy. Microscopic examination of the haematoxylin and eosin-stained anal condyloma tissue revealed koilocytic change, ulceration and brisk histiocytic inflammation containing numerous small intracellular bodies suggestive of *Leishmania* amastigotes. A bone marrow biopsy was performed and demonstrated similar intracellular forms. Anal condyloma tissue and bone marrow aspirate were sent to the Centers for Disease Control and Prevention's Parasitic Diseases Branch for confirmation of *Leishmania* and speciation. Specific immunohistochemical staining for *Leishmania* in the tissue section was positive and the species was confirmed as *Leishmania donovani* by PCR. Subsequently, the patient resumed highly active antiretroviral therapy and received anti-*Leishmania* therapy.

**Conclusion:**

Whilst the presentation of VL in HIV-positive patients is often similar to those without HIV, here we describe an unusual initial presentation of leishmaniasis in an HIV-positive patient where the parasite was found in an anal condyloma. VL is a critical diagnosis that should be considered and pursued when leishmaniasis is encountered in seemingly illogical clinical settings.

## Introduction

Leishmaniasis is not commonly encountered in the USA; however, the parasite is endemic in tropical and subtropical regions, and it is estimated that 200 million people worldwide are at risk of acquiring the infection. The parasite is transmitted through the bite of female phlebotomine sandflies; infectious promastigotes are injected into the skin and are subsequently taken up by professional phagocytic cells. Promastigotes next undergo a developmental switch to the ovoid, non-flagellated amastigotes inside the macrophages. The parasite multiplies as an amastigote in macrophages and other cell types, and is subsequently taken up by a sandfly blood meal. In the sandfly, the amastigotes revert to promastigotes, multiply in the insect midgut and migrate to the proboscis to infect the mammalian host during the next blood meal.

The genus *Leishmania* contains 30 species, 21 of which infect mammals, including humans. The signs and symptoms of leishmaniasis vary based upon the infecting species and the host immune status. Infection may result in cutaneous, or mucocutaneous disease which is most likely to be encountered in dermatopathology specimens, or in visceral disease. Cutaneous disease is most commonly associated with a focal ulcers with underlying infection directly at the sandfly bite site, which often heals without intervention. Mucocutaneous disease describes spread to the mucosa; mucosal lesions fail to heal spontaneously and secondary bacterial infections may be fatal. Visceral leishmaniasis (VL) may be asymptomatic to severe, and frequently presents with fever, malaise, anorexia, hepatomegaly, splenomegaly and lymphadenopathy. Due to the variety of signs and symptoms, leishmaniasis may be mistaken for other diseases, especially outside of endemic areas. We present a case of VL which was first identified in an anal condyloma. This location is very unusual and has not been previously described in the literature.

## Case report

The patient is a 24-year-old human immunodeficiency virus (HIV)-positive male from Nicaragua who presented for excision of condylomas on his anal mucosa. He was diagnosed with HIV/AIDS ∼3 years earlier and was started on antiretroviral therapy at that time. At the time of his diagnosis with HIV/AIDS, a peri-rectal skin tag was also noted, but no other symptoms or cutaneous findings were observed. At 1 month prior to his excision of his condyloma, the patient had an HIV viral load of 65 copies ml^− 1^ and CD4 T cell count of 144 cells μl^− 1^. At the time of the procedure the patient had no additional health concerns, cutaneous findings or symptoms, but he was noted to have been only intermittently compliant with his antiretroviral therapy due to intolerance of side-effects and lack of medical insurance.

### Investigations

**Fig. 1:**
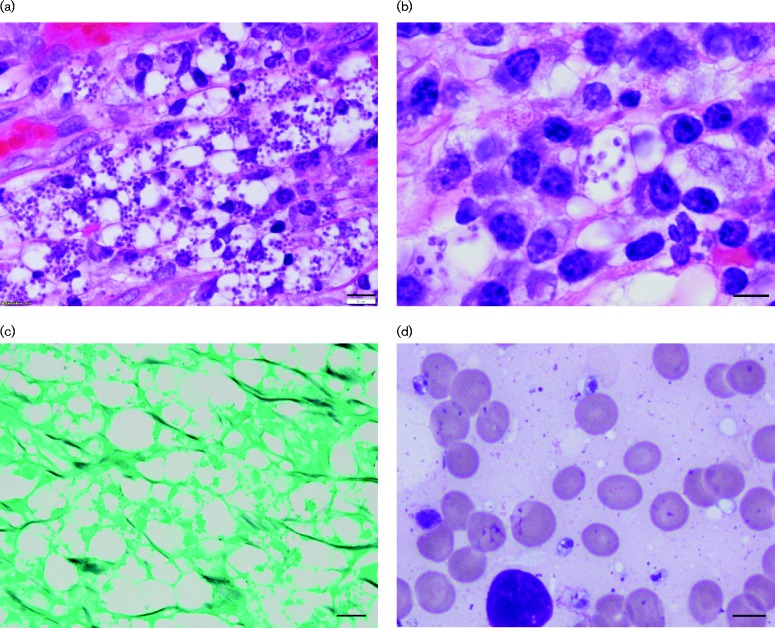
(a) Haematoxylin and eosin stain of rectal condyloma tissue revealed multiple intracellular forms with an occasional kinetoplast ( × 500; bar, 10 μm). (b) Inset of (a) ( × 500; bar, 5 μm). (c) Grocott–Gomori methenamine silver stain for fungi was negative ( × 500; bar, 10 μm). (d) Giemsa stain of bone marrow also revealed *Leishmania* amastigotes with characteristic kinetoplast ( × 1000; bar, 5 μm).

Anal condyloma tissue was submitted for histopathology. Microscopic examination of the haematoxylin and eosin-stained anal condyloma tissue revealed koilocytic change, ulceration and brisk histiocytic inflammation containing numerous small intracellular bodies with kinetoplasts, suggestive of *Leishmania* amastigotes (cutaneous herpesvirus cytopathic effect was also present) (Fig. 1a, b). Grocott–Gomori methenamine silver (Fig. 1c), Periodic Acid–Schiff, mucicarmine and Fontana–Masson stains were negative (not shown), which indicated the intracellular objects were not fungal in origin. The patient also had a few papillary lesions on his back, which were biopsied and also contained forms consistent with *Leishmania* species amastigotes. Given the unusual anatomical location (i.e. anal mucosa) for a disease transmitted by an insect vector coupled with the patient's previous residence in Central America, concerns for VL arose. The patient had lived in the USA for the previous 6 years and had no travel history to areas endemic for *Leishmania* since leaving Nicaragua. A bone marrow biopsy was performed and demonstrated similar intracellular forms by Giemsa stain (Fig. 1d), confirming that the patient did have VL.

The condyloma tissue and bone marrow aspirate were sent to the Centers for Disease Control and Prevention (CDC)’s Parasitic Diseases Branch for confirmation of *Leishmania* infection and speciation. Specific immunohistochemical staining of the tissue section was positive for *Leishmania* species. *Leishmania* reverse transcription PCR was positive, and *Leishmania* PCR and DNA sequencing identified the isolate as *Leishmania donovani*. Culture isoenzyme results also matched *L. donovani*.

### Treatment

Accordingly, the patient resumed antiretroviral therapy and received anti-*Leishmania* therapy (intravenous Pentostam) in coordination with the CDC. The lesions on the patient's back diminished in size and a second bone marrow biopsy was negative by microscopy, but did grow parasites upon culture.

### Outcome and follow-up

The patient has completed anti-*Leishmania* treatment and is stable on antiretroviral therapy.

## Discussion

Anal and rectal condylomas are caused by human papilloma virus (HPV), a sexually transmitted infection. Due to shared risk factors, HIV and HPV co-infections are common. Notably, HIV-positive individuals are more likely to develop symptomatic HPV infection. Whilst the disease severity corresponds to the level of immunosuppression, antiretroviral therapy has not reduced the incidence of HPV-associated carcinomas (Palefsky, 2009). The bivalent vaccine (Cervarix) offers protection against high-risk HPV genotypes (HPV16 and 18) associated with cancer, and the quadrivalent vaccine (Gardasil) offers protection against the same high-risk HPV genotypes and a few prevalent strains associated with genital warts (HPV6 and 11) (Dochez *et al.*, 2014). However, these vaccines do not provide protection against the numerous other strains of HPV and patients should be instructed on the necessity of safe sex practices to prevent infection.

The incidence of HIV and *Leishmania* co-infection is on the rise. Whilst HIV is more prevalent in urban regions and *Leishmania* in rural regions, increased urbanization and travel is presumed to contribute to the rate of co-infection (Saporito *et al.*, 2013). HIV-positive individuals, especially those with CD4 T-cell counts < 200 cells μl^− 1^, have a much greater chance of developing VL (Jarvis & Lockwood, 2013). HIV-positive patients with VL typically have similar signs as HIV-negative VL patients (Jarvis & Lockwood, 2013; Saporito *et al.*, 2013).

Cutaneous dissemination of VL may present as a pigmented or erythematous papule, lesion, rash or nodule (Balkhair & Ben Abid, 2008; Calza *et al.*, 2004; González-Beato *et al.*, 2000; Santos-Oliveira *et al.*, 2011). As the parasites replicate in macrophages, atypical cutaneous manifestations of VL may occur where there is a cutaneous inflammatory process. For example, one patient presented with conjunctival squamous cell carcinoma and another with dermatofibroma-like lesions (Bielory *et al.*, 2011; Forsyth *et al.*, 2003). *Leishmania* parasites have also been detected in tattoos and herpes zoster lesions (Lopez-Medrano *et al.*, 2007; Colebunders *et al.*, 1999). To the best of our knowledge, this is the first report of leishmaniasis presenting in an anal condyloma. It is unlikely that the parasites in the condyloma represent the site of initial cutaneous infection. It is more likely that VL occurred as reactivated disease upon HIV-induced immunosuppression.

HIV and *Leishmania* species both infect cells of the monocyte lineage. Previous investigations have shown these micro-organisms to be synergistically pathogenic. HIV co-infection of macrophages enhances *Leishmania* uptake and intracellular replication. Furthermore, *Leishmania* infection promotes the growth and survival of HIV-infected monocytes, which stimulates HIV replication. This results in higher rates of VL treatment failure, relapse and case fatality compared with HIV-negative VL patients (Jarvis & Lockwood, 2013). These findings highlight the importance of thoroughly investigating all suspicious signs found in an immunocompromised patient and maintaining an awareness for the possibility of *Leishmania* infection, especially in patients from endemic areas.
